# One-Pot Fabrication of Ginger-Waste-Derived Ionic Liquid Electrospun Films: An Efficient Preparation Strategy with Enhanced Antibacterial Functionality

**DOI:** 10.3390/foods14061058

**Published:** 2025-03-20

**Authors:** Xingran Kou, Kangning Ma, Xin Huang, Hui Wang, Qinfei Ke

**Affiliations:** 1 Key Laboratory of Textile Science & Technology, Ministry of Education, College of Textiles, Donghua University, Shanghai 201620, China; 2 Collaborative Innovation Center of Fragrance Flavour and Cosmetics, Shanghai Institute of Technology, School of Perfume and Aroma Technology (Shanghai Research Institute of Fragrance & Flavour Industry), Shanghai 201418, China; mkn971009@163.com (K.M.); hx@sit.edu.cn (X.H.); wh1999laura@163.com (H.W.)

**Keywords:** ginger waste, cellulose, wet electrospinning, active food packaging

## Abstract

In the process of ginger deep processing, a lot of waste is generated which is rich in biopolymers and active ingredients such as cellulose, starch, gingerol, and gingerol, but its low utilization rate leads to waste of resources. In this study, ginger waste residue, cellulose, and bioactive substances were spun into fiber materials by wet electrospinning technology with 1-butyl-3-methylimidazole acetate ([Bmim]Ac) as solvent. Fiber plasticization and [Bmim]Ac removal were achieved by dynamic deionized water coagulation bath. Scanning electron microscopy (SEM) and tensile strength analysis showed that the obtained GC-1 and GC-2 films have a non-uniform diameter, with a clear fiber structure and strong tensile strength. Fourier transform infrared spectroscopy (FTIR) and X-ray diffraction (XRD) confirmed that cellulose transforms from type I to type II crystal structure, and [Bmim]Ac is effectively removed. The inhibition rate of 6-Shogaol-impregnated GC film against *Escherichia coli* and *Staphylococcus aureus* was 99%. The experiment of strawberry preservation verified the potential of GC film in food preservation. In this study, the high-value utilization of ginger waste in food packaging was realized by preparing antibacterial electrospun fiber films.

## 1. Introduction

Food packaging serves a pivotal role in the preservation of food, effectively inhibiting the proliferation of detrimental microorganisms, decelerating quality deterioration, and thereby prolonging the shelf life [1]. Antimicrobial film for active food packaging is a current research hotspot in the field of food packaging, both domestically and internationally [2,3,4]. It involves incorporating antimicrobial agents (inorganic, organic, and natural antimicrobial agents) into the film substrate to prepare packaging films with antimicrobial activity. Currently, food packaging film substrates are still dominated by petroleum-based plastics such as polyethylene, polypropylene, and polyethylene terephthalate; their sources are unsustainable, difficult to degrade, and have huge environmental and ecological hazards [5].

Biopolymers are large molecular compounds composed of natural substances found in living organisms, which include natural biopolymers such as proteins, polysaccharides, natural rubber, and lipids, as well as synthetic polymers derived from natural compounds, such as polylactic acid (PLA) [6,7,8]. Due to its excellent biocompatibility and biodegradability, biopolymers have extensive applications in the preparation of biodegradable films [1,9]. China has abundant natural spice resources, with large production and consumption quantities. However, the utilization rate of byproducts from deep processing is relatively low. Ginger, due to its high content of valuable gingerol resin and volatile oil, is widely used for processing and preparing the byproducts of ginger. However, gingerol resin and volatile oil in ginger account for only 4–10% of the total dry weight of the rhizome, resulting in a significant generation of ginger waste [10]. Ginger waste contains abundant natural biopolymers with high polymerization, good molecular orientation, stable chemical properties, and biodegradability, such as starch and cellulose, which urgently need to be recovered and utilized. Our previous research has revealed that the bioactive components in ginger, such as gingerol and shogaol, exhibit outstanding activities like antioxidant, anti-inflammatory, and antibacterial properties [11]. Therefore, gingerol and shogaol can also serve as natural antibacterial agents in food, medicine, textiles, cosmetics, and other fields. Currently, the primary disposal methods of dealing with ginger waste are still centered around disposal, burning, and utilization as animal feed, resulting in a low utilization rate. Developing an approach for the comprehensive utilization of biomass resources and bioactive substances in ginger waste is essential.

Among various film preparation techniques, nanofibers produced by electrospinning technology have shown significant potential in a wide range of applications due to their unique characteristics, such as high surface area, ultrafine structure of the obtained nanofibers, and biocompatibility [12]. Furthermore, electrospinning technology, as an efficient encapsulation strategy for bioactive substances, has gained widespread attention in the preparation of active food packaging materials [13,14]. It provides an array of advantages, including a high degree of encapsulation efficiency, versatile selection options for bioactive substances, as well as the capability to prolong the release profile of bioactive compounds [15,16]. In this study, electrospinning technology was employed to encapsulate the bioactive components in ginger waste with biopolymers such as cellulose and starch from ginger waste. Ginger waste is efficiently utilized to produce active food packaging materials.

To avoid the production process challenges associated with the direct use of ginger waste, this study utilized a mixture of ginger waste and cellulose as the electrospinning matrix. Ionic liquids, as a novel and efficient solvent for cellulose, are widely applied in the green processing and utilization of cellulose [17,18,19,20]. Previous research has shown that cellulose can be dissolved in ionic liquids and regenerated, resulting in nanofibers with excellent properties such as high surface area, large tensile strength, high Young’s modulus, and a high aspect ratio [21]. Furthermore, our research has revealed that ionic liquids can convert gingerol in ginger into shogaol, which exhibits stronger antibacterial activity [22]. In the study by Edvinas Krugly et al., three types of ionic liquids were used to dissolve cotton cellulose for wet electrospinning [23,24]. The fibers were then subjected to a three-stage washing process to remove the ionic liquids, resulting in various fiber morphologies. This research confirmed the feasibility of cellulose electrospinning in ionic liquids. Significantly, ionic liquids, when used as solvents, could be recycled and reused, significantly reducing the cost of the electrospinning process.

Therefore, this study aims to start with cellulose and ginger waste, using wet electrospinning technology with 1-butyl-3-methylimidazolium acetate ([Bmim]Ac) as the solvent. Fiber films with excellent antibacterial activity were successfully prepared using cellulose, ginger waste, and 6-Shogaol as raw materials. Using a dynamic coagulation bath of deionized water, [Bmim]Ac was thoroughly washed out from the fibers. The nanofiber film was characterized using SEM, XRD, FTIR, and a universal material testing machine to examine the microstructure, crystal structure, the elution effect of [Bmim]Ac, and mechanical properties. The antibacterial activity of the nanofiber against Escherichia coli and Staphylococcus aureus was assessed using a co-culture method. Finally, the application potential of ginger waste/cellulose powder fibers loaded with 6-Shogaol in fruit preservation was confirmed through strawberry preservation experiments.

## 2. Materials and Methods

### 2.1. Materials

Ginger was obtained from the Fengxian District Agricultural and Trade Market in Shanghai, China; cellulose was supplied by Shanghai Aladdin Biochemical Technology Co., Ltd. (Shanghai, China); 1-butyl-3-methylimidazolium acetate ([Bmim]Ac, CAS: 284049-75-8) was supplied by Lanzhou Greenchem ILs (Lanzhou, China); the strawberries were locally harvested in Fengxian District, Shanghai.

### 2.2. Biomass Components in Ginger Waste Residue

The content of various biomass components in ginger residues was roughly measured using an extraction method [25]. Ginger residues obtained by drying and grinding the residues left after juicing ginger were subjected to a process. Deionized water was added to the ginger residue powder at a material-to-water ratio of 1:30, stirred at 80 °C for 60 min, and this process was repeated three times. The mixture was then dried at 55–60 °C. The reduced mass was approximately the mass of water-soluble starch. The residue was degreased with petroleum ether and acetone. The degreased samples were treated with a sodium hypochlorite solution (50 g/L, pH = 4.0) at 70 °C for 2 h. The residue was washed and dried to obtain delignified ginger residues. The delignified ginger residues were treated with 10% potassium hydroxide at 55 °C for 12 h, filtered, and dried to remove hemicellulose. The resulting samples, after each step, were weighed to calculate the approximate content of each biomass component in the ginger residues.

### 2.3. Preparation of Electrospinning Solution

The waste residue obtained after ginger juice extraction is dried and pulverized to obtain ginger waste powder. Ginger waste/[Bmim]Ac solution (3 wt%) is prepared by adding ginger waste powder to [Bmim]Ac, followed by stirring at 90 °C for 5 h. Cellulose/[Bmim]Ac solution (10 wt%) is prepared by adding cellulose powder to the [Bmim]Ac solution and stirring at 90 °C for 5 h. The electrospinning solution was obtained by mixing cellulose/[Bmim]Ac solution (10 wt%) and ginger waste/[Bmim]Ac solution (3 wt%) with a certain mass ratio and stirring at 90 °C for 1 h. To explore the formation conditions of electrospun fibers from ginger waste/cellulose powder, we prepared six different polymer solutions, as shown in Table 1. Cellulose powder can completely dissolve in [Bmim]Ac. However, ginger waste powder cannot completely dissolve in [Bmim]Ac, which may be related to the complex composition of ginger waste. Even with an increased dissolution time, a small amount of solute remains undissolved, but it does not affect the formation of electrospinning jets. This study mainly used cellulose powder as the main substrate, and on the basis of keeping the added amount of ginger waste constant, the content of cellulose powder was reduced, and the proportion of ginger waste showed an increasing trend.

Furthermore, to enhance the bioactivity of ginger waste/cellulose powder fibers, we added 5 wt% 6-Shogaol (based on the mass of cellulose powder) to the GC-1 solution, yielding the 6-Shogaol-GC-1 solution.

### 2.4. Viscosity and Conductivity

A viscosimeter (ROTAVISC hi-vi, IKA Company, Staufen im Breisgau, Germany) was used to measure the viscosity of the electrospun solution at 100 rpm, 40 °C; The electrical conductivity of electrospinning solution was measured at 40 °C by a bench conductivity meter (FE38, Mettler-Toledo International Inc., Columbus, OH, USA).

### 2.5. Electrospinning

The electrospinning solution was added to a 10 mL syringe and the syringe was secured onto the syringe pump of the electrospinning machine. The parameters of the electrospinning machine were set as follows: solution flow rate at 0.08 mL/min, collection distance at 5–10 cm, collector roller speed at 2 rpm/min, temperature inside the electrospinning machine at 40 °C, and electrospinning voltage at 20–28 kV. During the electrospinning process, the lower half of the collecting roller was immersed in the coagulation bath. After electrospinning, the electrospun fibers were air-dried at room temperature for 48 h.

The main purpose of the coagulation bath is to elute the [Bmim]Ac from the fibers, facilitating the plasticization of ginger waste/cellulose powder fibers. Firstly, we used deionized water and an ethanol–water solution as coagulation baths, respectively, to investigate the effect of coagulation bath type on the removal of ionic liquid from electrospun fibers of ginger waste/cellulose powder. In addition, Edvinas Krugly et al. carried out three stages of elution on the cotton cellulose fiber after electrospinning, and the ionic liquid in the fiber was completely removed [24]. Reducing the concentration of the ionic liquid in the coagulation bath or elution solution can create a larger concentration gradient between the coagulation bath or elution solution and the electrospun fibers. This facilitates better removal of the ionic liquid from the electrospun fibers.

**Figure 1:**
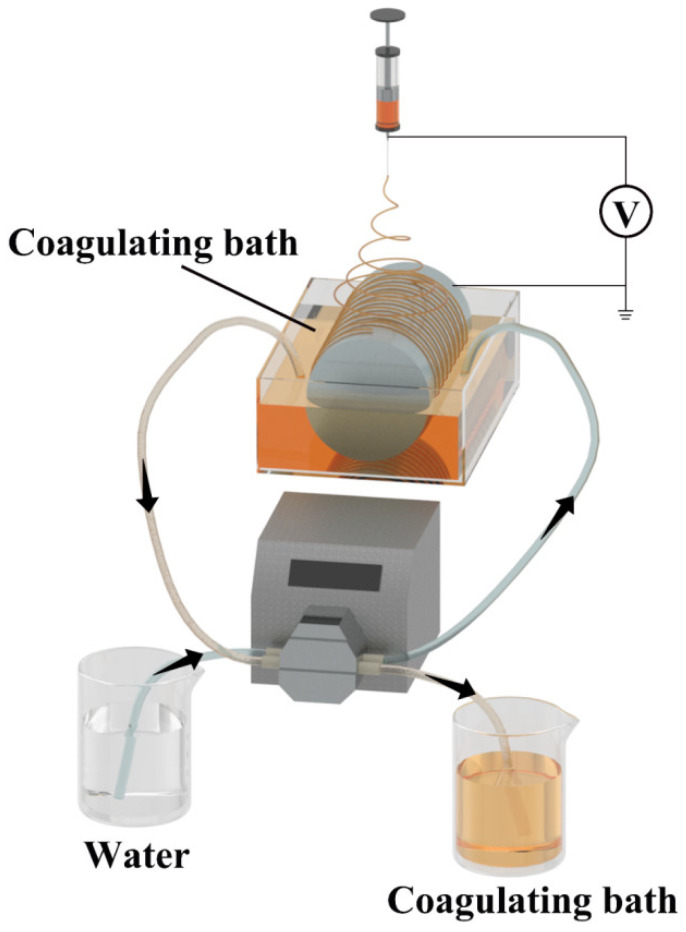
Schematic diagram of electrospinning setup.

Therefore, we added a peristaltic pump to the coagulation bath to achieve dynamic circulation of the deionized water. In short, the elution solution containing ionic liquid in the coagulation bath is continuously discharged, while deionized water is replenished into the coagulation bath, as show in Figure 1. The concentration of the ionic liquid in the coagulation bath remains at a relatively low level, ensuring the efficient and complete elution of the ionic liquid from the electrospun fibers of ginger waste/cellulose. The speed of the peristaltic pump is 100 rpm, delivering approximately 1.7 L/h. The electrospinning results of six different polymer solutions have been presented in Table 1, and the next experiment will be conducted according to whether the spinning can be successful for screening and preparation.

### 2.6. Morphology

The morphology of ginger waste/cellulose powder fibers was characterized by scanning electron microscopy (Sigma 300, Carl Zeiss AG, Oberkochen, Germany).

### 2.7. The Elution Effect of [Bmim]Ac

By recording the attenuated total reflectance–Fourier transform–infrared (FTIR) spectra (Spectrum Two, Perkin Elmer, Waltham, MA, USA) in the range of 4000~500 cm^−1^, potential chemical changes in cellulose and ginger waste were observed. In addition, the residue of [Bmim]Ac in ginger waste/cellulose powder fibers can also be screened in the FTIR spectra, verifying the effectiveness of the coagulation bath in elution.

### 2.8. Crystal Structure Change of Cellulose

The crystallinity of the cellulose samples was analyzed using an X-ray diffractometer (D8 Advance, Bruker, Bremen, Germany). The X-ray source was Cu-Kα, the test current was set as 50 mA, the voltage was set as 40 kV, the DS/SS was set as 0.5, and the scanning angle ranged from 5° to 50° with the scanning rate of 8°/min.

### 2.9. Mechanical Property

The tensile strength (TS) of the film samples was measured using an intelligent electronic tensile testing machine (C610M, Labthink, Jinan, China). The initial grip distance was 30 mm, and the speed was 3 mm/s. Before testing, the film was cut into rectangular strips (60 mm × 10 mm). Three strips were tested for each film sample.

The formula for calculating TS is as follows:

(1) $$TS\left(MPa\right)=\frac{F_{m}}{d\times b}$$

In the formula, *F_m_* represents the maximum tensile force sustained by the film sample (N); *d* is the width of the film (mm); *b* is the thickness of the film (mm).

### 2.10. The Determination of Compound Concentration Determination

A standard solution of 6-Shogaol was prepared by dissolving 6-Shogaol standard in methanol to obtain concentrations of 5 mg/mL, 2.5 mg/mL, 1 mg/mL, 0.5 mg/mL, and 0.1 mg/mL. A standard curve for 6-Shogaol concentration was established using HPLC (RF-20A, Shimadzu Global Laboratory Consumables Co., Ltd., Kyoto, Japan) with a mobile phase of 80% acetonitrile and 20% water at a total flow rate of 1 mL/min. For the sample, 200 mg of 6-Shogaol-GC-1 fiber was added to 2 mL of methanol. After thorough shaking, the mixture was allowed to stand for 1 h. The content of 6-Shogaol in 6-Shogaol-GC-1 electrospun fiber was determined using HPLC and the standard curve for 6-Shogaol concentration.

### 2.11. Antibacterial Activity

The antibacterial activity of 6-Shogaol-loaded ginger waste/cellulose electrospun fibers was evaluated using a co-culture method. *Escherichia coli* (*E. coli*) and *Staphylococcus aureus* (*S. aureus*) solutions were diluted to approximately 10^8^ CFU/mL. Measures of 5 mL of bacterial solution were separately added to 100 mg and 150 mg of the 6-Shogaol-loaded ginger waste/cellulose electrospun fibers. The mixture was incubated at 37 °C with agitation at 200 rpm for 3 h. After cultivation, 200 μL of the bacterial suspension was spread evenly on a nutrient agar plate, followed by incubation at 37 °C for 24 h. The bacterial colonies on the agar plate were recorded and counted to calculate the antibacterial rate. The ginger waste/cellulose electrospun fibers served as the control group, and the bacterial solution without any fibers served as the blank control group. The formula for calculating the antibacterial rate is as follows:

(2) $$Antibacterial\;activity\left(\%\right)=\frac{A_{0}-A_{t}}{A_{0}}\times 100\%$$

*A*_0_ is the number of colonies in the blank control group, and *A_t_* is the number of colonies in the experimental group or control group.

### 2.12. Strawberry Preservation

The method was slightly modified on the basis of Kou et al. [26]. Eighteen strawberries were selected from the freshly picked batch, ensuring they had a good appearance and were free from any damage. The strawberries were randomly divided into three groups and placed in plastic storage containers, with each group containing six strawberries. These were labeled as the experimental group, control group, and blank control group, respectively. Experimental group strawberries were coated with 6-Shogaol-loaded ginger waste/cellulose electrospun fibers, the control group strawberries were coated with ginger waste/cellulose electrospun fibers, and the blank control group strawberries received no treatment. All the preservation boxes were placed in a constant-temperature and -humidity incubator at 25 °C with 60% relative humidity. The daily changes in the mass of strawberries were measured and the external appearance changes were observed. The mass loss rate of strawberries during storage was calculated using the formula for mass loss rate:

(3) $$Wr\left(\%\right)=\frac{M_{o}-M_{t}}{M_{0}}\times 100\%$$

where W_r_ represents the rate of mass loss of the strawberries during storage, *M*_0_ represents the quality of the strawberries on day 1, and *M_t_* represents the quality of the strawberries on day *t*.

### 2.13. Degradation Analysis

Plastic cups (500 mL) with common garden soil (total soil depth was 10 cm) were prepared, and dry film samples (3 cm × 3 cm) were buried 3 cm below the surface of the soil. The samples were sprayed with 20 mL of tap water using a small watering can every day to keep the soil moisture at 40%, and the degradation degree of tested samples were observed.

### 2.14. Statistical Analysis

All experiments were conducted with a minimum of three replicates, and the results are expressed as the mean ± standard deviation (M ± SD). Statistical analysis was performed using analysis of variance (ANOVA) and Tukey’s multiple range test, with a significance level set at *p* < 0.05. The obtained results were graphically presented using OriginPro 8.5.0 software (OriginLab, Northampton, MA, USA).

## 3. Results and Discussion

### 3.1. Biomass Components in Ginger Waste Residue

**Figure 2:**
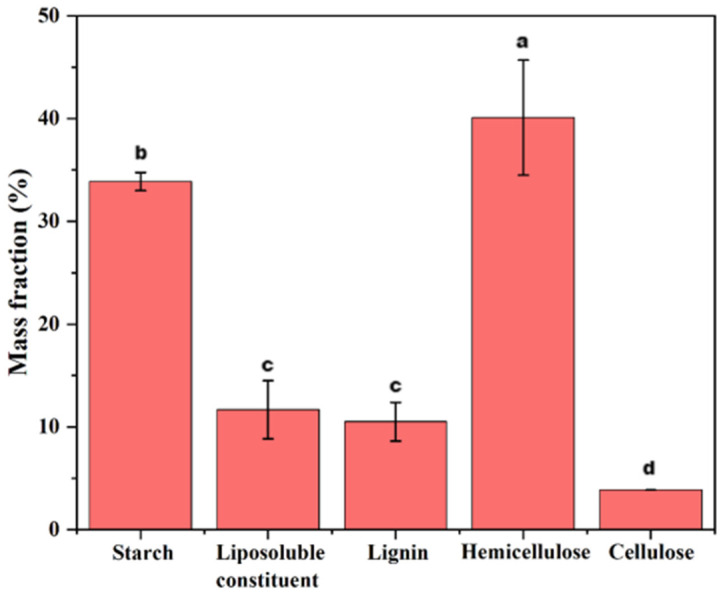
Mass fraction of biomass in ginger waste residue. Different letters on columns indicated a significant difference (*p* < 0.05).

An analysis of the biomass components in ginger waste was conducted using an extraction method, with the results presented in Figure 2. The analysis revealed that ginger waste contains a relatively high proportion of water-soluble starch (33.9%) and hemicellulose (40.1%). In contrast, lipid-soluble components, lignin, and cellulose were present in lower proportions, accounting for 11.6%, 10.5%, and 3.9%, respectively. This method provided an initial quantitative assessment of the biomass composition in ginger waste.

### 3.2. Elaboration Conditions of Electrospinning Solution

The preparation involved testing different concentrations of biopolymers and various electrospinning conditions, ultimately resulting in the production of electrospun fibers from ginger waste/cellulose. Among them, the GC-1 and GC-2 solutions exhibited the best spinnability, with the optimal spinning voltage being 25.5 kV. The electrospun solutions of GC-3 and GC-4 showed certain spinnability, but their jets were unstable during the electrospinning process. Electrospinning of cellulose in ionic liquids has been widely studied. However, the focus of this study was to incorporate ginger waste into the solution and use a “one-pot” approach to prepare ginger waste/cellulose electrospun fibers. Therefore, this study provided a solution for the reuse of ginger waste, which could be used to prepare active food packaging.

### 3.3. Viscosity and Conductivity

The viscosity and conductivity of the electrospinning solution were measured using a viscometer and a conductivity meter, and the results were shown in Table 2. GC-1, GC-2, GC-3, and GC-4 electrospinning solutions exhibited decreasing viscosity and increasing conductivity in sequence. This was related to the polymer concentration in the solution. Compared to other electrospinning solutions, GC-1 had a higher polymer concentration and lower ionic liquid concentration, resulting in higher viscosity and lower conductivity, which was consistent with the study of Araldi da Silva et al. [17].

### 3.4. Morphology

**Figure 3:**
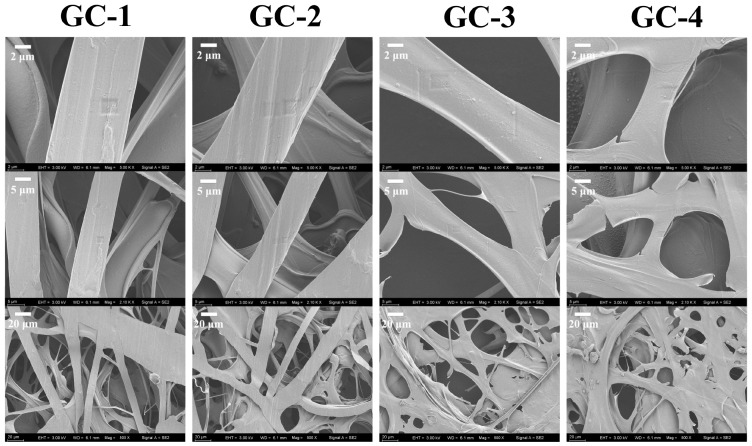
SEM images of ginger waste/cellulose electrospun fiber.

The SEM images of electrospun fibers from ginger waste/cellulose powder are shown in Figure 3. The plasticization process of fibers involved the continuous transfer of the ionic liquid to the coagulation bath, which might result in an uneven surface of the fibers [27]. Additionally, due to the complex polymer composition in ginger waste powder, a homogeneous electrospinning solution could not be formed. As a result, the fibers formed are not uniform in diameter. Due to the excellent spinnability of GC-1 and GC-2 solutions, a clearer fiber structure was eventually formed. As the spinnability of the electrospinning solution decreases, the GC-4 solution formed fibers with film inserts.

### 3.5. FTIR

The FTIR images showed that the main chemical components of ginger waste and cellulose remain unchanged during the dissolution and regeneration process in [Bmim]Ac. The peak at 1428 cm^−1^ was assigned to -CH_2_ scissoring motion, which was related to the crystallinity of cellulose. Compared with cellulose powder, the strength of this band in regenerated cellulose was reduced, indicating a decrease in crystallinity. The peak associated with the amorphous region of cellulose was located at 895 cm^−1^. The peak was sharper in this spectrum of electrospun fiber of ginger waste/cellulose. This phenomenon indicated that, during the dissolution and regeneration process in [Bmim]Ac, cellulose I was transformed into cellulose II [28].

In wet electrospinning using ionic liquids as solvents, the efficiency of ionic liquid removal significantly affected the plasticization of fibers and their morphology. The peak observed in the electrospun fiber at 1580–1550 cm^−1^ was assigned to the stretching vibrations of the imidazole ring [24]. Therefore, the degree of elution of [Bmim]Ac from the fibers could be determined by observing the presence of the peak at this location. After washing with a deionized water coagulation bath, a distinct peak in the region of 1567 cm^−1^ was still present, indicating the residual presence of [Bmim]Ac in the electrospun fibers of ginger waste/cellulose powder. When using 20% ethanol, 40% ethanol, and 60% ethanol as coagulation baths, the peak around 1567 cm^−1^ in the region disappeared. This indicated that the ethanol solution is more effective than deionized water in eluting [Bmim]Ac, enabling the complete removal of [Bmim]Ac from the fibers. However, using an ethanol solution as the coagulation bath not only incurred economic costs but also extracted bioactive components from ginger waste/cellulose electrospun fibers. Therefore, in this study, a dynamic coagulation bath of deionized water was employed to elute [Bmim]Ac from ginger waste/cellulose electrospun fibers. The disappearance of the peak around 1567 cm^−1^ in the region indicated that the [Bmim]Ac in the fibers was completely eluted. The dynamic coagulation bath could maintain a high concentration gradient of ionic liquid between the coagulation bath and the fiber, so as to improve the elution efficiency of ionic liquid in nanofibers, which was consistent with the study of Lassi V. Tiihonen et al. [29].

### 3.6. XRD

**Figure 4:**
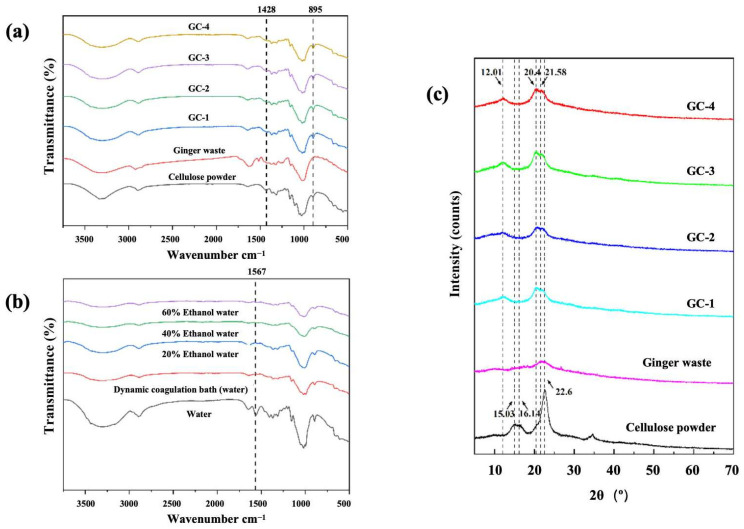
(**a**) FTIR spectra of different ginger waste/cellulose electrospun fiber samples; (**b**) FTIR spectra of ginger waste/cellulose electrospun fiber at a variety of elution conditions; (**c**) XRD spectra of ginger waste/cellulose electrospun fiber samples.

Cellulose had two main crystalline structures, namely I-type crystal and II-type crystal. The characteristic diffraction peak of cellulose I crystalline structure was around 2θ = 22.6°, while that of cellulose II crystalline structure was around 2θ = 20.8°. As shown in Figure 4, the XRD spectrum of the original cellulose powder exhibited characteristic peaks around 2θ = 15.03°, 16.14°, and 22.6°. In contrast, the XRD spectrum of cellulose fibers treated with ionic liquid showed characteristic peaks around 2θ = 12.01°, 20.4°, and 21.58° [30]. Therefore, it could be inferred that the crystalline structure of cellulose undergoes a transformation during the process of dissolving in the ionic liquid, shifting from the I-type crystal structure to the II-type crystal structure. This result was consistent with that of FTIR.

### 3.7. Mechanical Property

**Figure 5:**
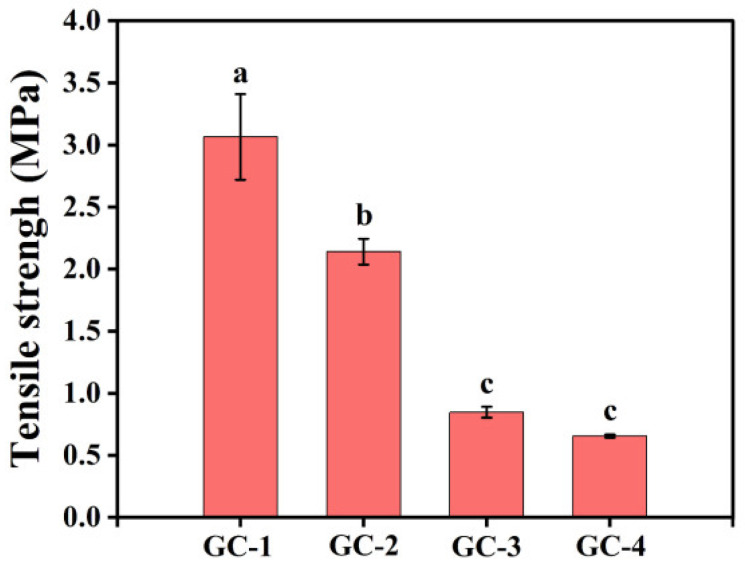
Tensile strength of different ginger waste/cellulose electrospun fiber samples. Different letters on columns indicated a significant difference (*p* < 0.05).

The tensile strengths of the GC-1, GC-2, GC-3, and GC-4 fibers are shown in Figure 5. The tensile strengths of the GC-1, GC-2, GC-3, and GC-4 fibers decreased sequentially, which may be related to the structure of the fibers. During the electrospinning process, the jets of GC-1 and GC-2 solutions were relatively stable, forming electrospun fibers with a clear structure and tight connections. As the spinnability of the electrospinning solution decreases, fibers with film inserts were formed. The connections between fibers with film inserts were weaker, leading to a decrease in the tensile strength of the fibers.

### 3.8. The Determination of Compound Concentration Determination

By conducting high-performance liquid chromatography (HPLC) analysis on 6-Shogaol standard solutions of various concentrations, a standard curve correlating the 6-Shogaol concentration (mg/mL) with the peak area was obtained. y=5160756.15x+249589.27 (R^2^ = 0.999887636). The peak area for the electrospun fibers of 6-Shogaol-loaded ginger waste/cellulose powder sample was 906,009, and according to the standard curve, the concentration was determined to be 0.1272 mg/mL. The lower load of 6-Shogaol was within our expectations. In our previous studies, we found that 6-Shogaol has some solubility in ionic liquid solutions. Therefore, a significant portion of 6-Shogaol in the fibers was lost during the elution process.

### 3.9. Antibacterial Activity

**Figure 6:**
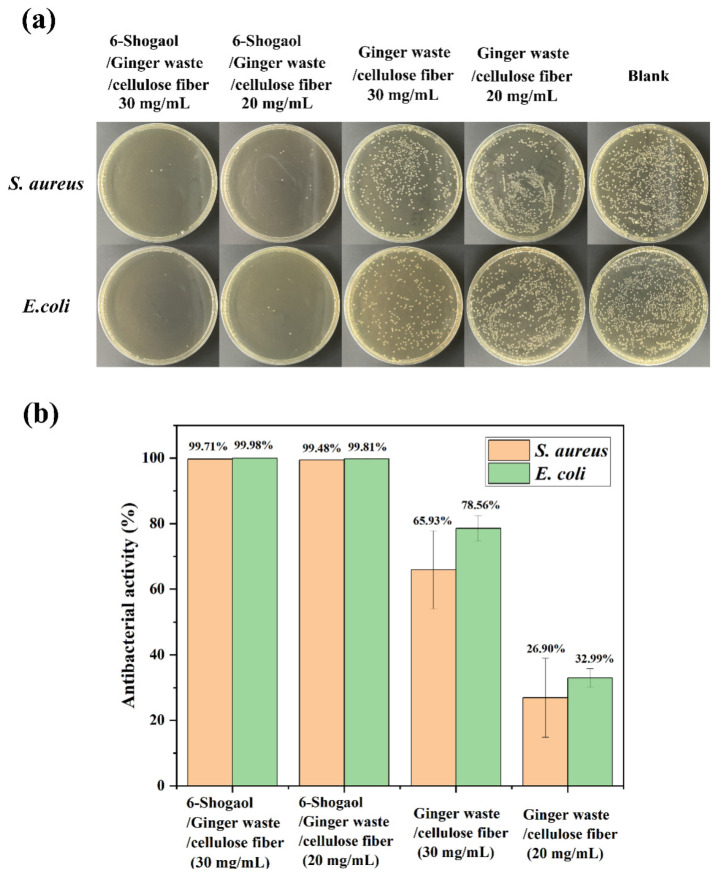
The antimicrobial activity of GC-1 and 6-Shogaol-GC-1 against *S. aureus* and *E. coli* was evaluated using a co-culture method. (**a**) Colony-forming units (CFUs); (**b**) antibacterial rate of GC-1 and 6-Shogaol-GC-1 against *S. aureus* and *E. coli*.

The antibacterial activity of different film samples against Gram-positive bacteria (*S. aureus*) and Gram-negative bacteria (*E. coli*) was evaluated, and the antibacterial results were shown in Figure 6. The electrospun fibers of ginger waste/cellulose powder exhibited certain antibacterial effects against *S. aureus* and *E. coli*, and the antibacterial activity was enhanced with the increase in fiber film concentration (mg/mL). The antibacterial activities (%) of electrospun fibers of ginger waste/cellulose powder against *S. aureus* and *E. coli* were 65.93% (30 mg/mL), 26.90% (20 mg/mL), 78.56% (30 mg/mL), and 32.99% (20 mg/mL), respectively. The addition of 6-Shogaol significantly enhanced the antibacterial activity of electrospun fibers of ginger waste/cellulose powder. Specifically, the antibacterial activities (%) of 6-Shogaol/ginger waste/cellulose powder electrospun fibers against S. aureus and E. coli were 99.70% (30 mg/mL), 99.48% (20 mg/mL), 99.98% (30 mg/mL), and 99.81% (20 mg/mL), respectively. 6-Shogaol is a hydrophobic compound capable of inserting itself into the lipid bilayer of microbial cell membranes, destroying their integrity [31]. This destruction leads to increased membrane permeability, leakage of cell contents, and ultimately cell death. In addition, 6-Shogaol can inhibit key enzyme activity of bacteria and interfere with their energy metabolism and substance synthesis [32]. For example, 6-Shogaol inhibits ATP synthesis in bacteria, resulting in insufficient energy supply. As a natural active ingredient, 6-Shogaol has a multi-target antimicrobial mechanism and is safer and more environmentally friendly than traditional preservatives, which usually exert antibacterial action through a single or few targets. However, the application of 6-Shogaol still requires further research on its stability, cost, and feasibility for large-scale production.

### 3.10. Application of the Ginger Waste/Cellulose Electrospun Fibers in Food Packaging

**Figure 7:**
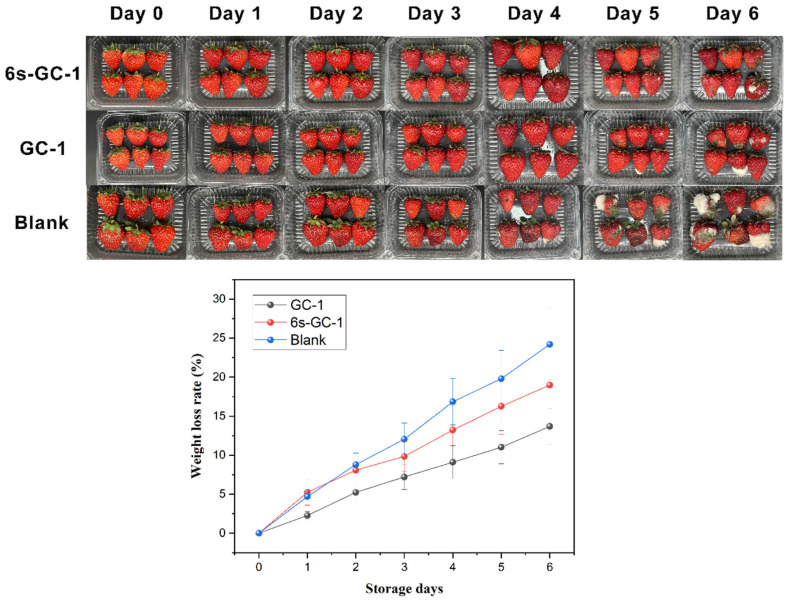
The weight loss rate and visual appearance changes of strawberries.

In order to observe the effect of ginger residue/cellulose electrospinning fiber on food preservation, strawberries were stored at 25 ± 1 °C. As shown in Figure 7, strawberries without fiber coverage served as the blank control group, while strawberries covered with GC-1 and 6-Shogaol-GC-1 were designated as the control group and experimental group, respectively. The weight loss rate and visual appearance changes of strawberries covered with the film were tested and observed.

All strawberries experienced varying degrees of weight loss during the storage period. Among them, strawberries without fiber coverage had the highest weight loss rate, while those covered with GC-1 had the lowest weight loss rate. This was because strawberries have a high water content, and their transpiration and respiration processes lead to moisture loss [33]. The fiber film coverage impeded the evaporation of moisture in the strawberries. In addition, strawberries without fiber coverage began to show mild signs of decay on the third day. GC-1 and 6-Shogaol-GC-1 started to exhibit varying degrees of decay on the fourth and fifth days, respectively. On the sixth day, the strawberries without fiber coverage had a decay rate of 100%, and the decay was severe, while the fiber-covered strawberries showed less severe decay.

Combined with changes in the rate of weight loss and visual appearance of strawberries, it could be seen that the covering of GC-1 and 6-Shogaol-GC-1 fibers slows the weight loss and decay of strawberries during storage. It could be seen that the fiber has potential application value in fruit preservation. In addition, according to the high-performance liquid chromatography test, the content of 6-gingerenol in the packaging film is 0.1272 mg/mL, which is far lower than the concentration of 6-gingerenol in harvested *roscoe* var. (1.85 mg/g), which reduces the safety risks in food packaging [34].

### 3.11. Film Degradability

**Figure 8:**
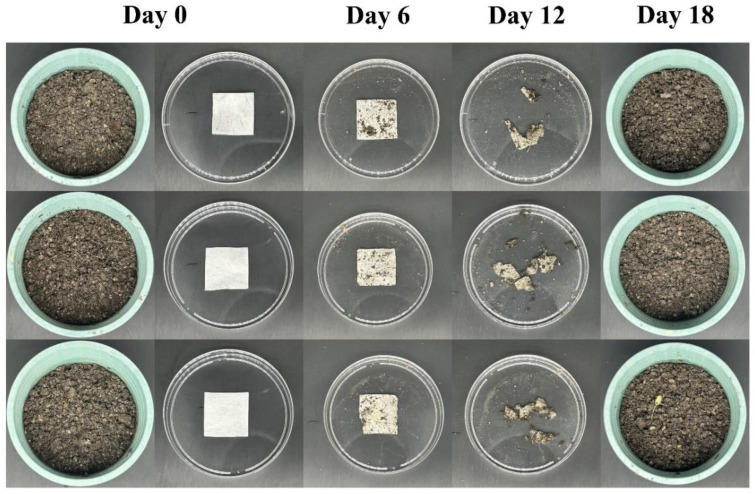
The weight loss rate and visual appearance changes of strawberries.

The main biomass components of GC-1 and 6-Shogaol-GC-1 ginger waste residue/cellulose electrospinning fiber membranes are the same. Therefore, the soil degradation experiment of GC-1 ginger waste residue/cellulose electrospinning fiber membranes was conducted to confirm the biodegradability of GC-1 and 6-Shogaol-GC-1 ginger waste residue/cellulose electrospinning fiber membranes. The experimental results of soil degradation of GC-1 ginger waste residue/cellulose electrospun fiber film are shown in Figure 8. The results of three parallel experiments showed that, on the 6th day of the experiment, the change of GC-1 ginger waste residue/cellulose electrospinning fiber membrane was not obvious; on the 12th day of the experiment, GC-1 ginger waste residue/cellulose electrospinning fiber membrane was partially degraded; on the 18th day of the experiment, there was no obvious GC-1 ginger waste residue/cellulose electrospinning fiber fragments in the horticultural soil, so it was considered that GC-1 ginger waste residue/cellulose electrospinning fiber was completely degraded. The soil degradation experiment confirmed the biodegradability of GC-1 and 6-Shogaol-GC-1 ginger waste residue/cellulose electrospinning fiber membranes.

## 4. Conclusions

This study utilized ginger waste and cellulose powder as raw materials, [Bmim]Ac as a solvent, and employed a wet electrospinning technique to fabricate ginger waste/cellulose electrospun fibers. The results indicated that GC-1 and GC-2 exhibit good spinnability, enabling the production of ginger waste/cellulose electrospun fibers with a well-defined fiber structure. Analysis of the characteristic peaks of imidazole ions at 1580–1550 cm^−1^ in the FTIR spectrum validated the thorough removal of [Bmim]Ac from ginger waste/cellulose electrospun fibers achieved through dynamic coagulation baths. Antibacterial tests indicated that ginger waste/cellulose electrospun fibers inhibit the growth of *S. aureus* and *E. coli*, and electrospun fibers loaded with 0.1272 wt% 6-Shogaol exhibited a high antibacterial rate of 99% against both bacteria. Strawberry preservation experiments demonstrated that 6-Shogaol/ginger waste/cellulose electrospun fibers effectively delay the decay of strawberries, showing potential applications in the field of food packaging. Additionally, this study proposed a novel approach for the recycling of ginger waste, providing new insights into the reuse of waste generated from spice processing.

## Figures and Tables

**Table 1:** Polymer concentration in [Bmim]Ac used to fabricate nanofibers from ginger waste and cellulose powder.

	3 wt% Ginger Waste/[Bmim]Ac (g)	10 wt% Cellulose Powder/[Bmim]Ac (g)	Polymer Concentration (wt%)	Result
GC-0	0	10	10	No jet formed
GC-1	1	10	9.36	Jet formed
GC-2	1	9	9.3	Jet formed
GC-3	1	8	9.22	Jet formed
GC-4	1	7	9.13	Jet formed
GC-5	1	4	8.6	No jet formed

**Table 2:** Viscosity and conductivity of electrospinning solution.

	Temperature (°C)	Viscosity (mPa·s)	Conductivity (μs/cm)
GC-1	40	22,693 ± 136	1429 ± 3
GC-2	40	22,120 ± 408	1465 ± 22
GC-3	40	21,080 ± 312	1480 ± 17
GC-4	40	19,493 ± 264	1732 ± 28

## Data Availability

The original contributions presented in the study are included in the article, further inquiries can be directed to the corresponding author.
